# Supplementary material for: A Systematic Review of the Development and Psychometric Properties of Loneliness Measures for Children and Adolescents
Source: Int J Environ Res Public Health. 2021 Mar 22;18(6):3285. doi: 10.3390/ijerph18063285 (PMC8004998; doi:10.3390/ijerph18063285)
Supplement: Supplementary file 1 [file ijerph-18-03285-s001.pdf]

International Journal of
Environmental Research and Public Health

Review

A Systematic Review of the Development and Psychometric Properties of Loneliness Measures for Children and Adolescents

Aimée Cole, Caroline Bond, Pamela Qualter and Marlies Maes

Supplementary Material

Table S1: Record of checklists from which Critical Appraisal statements were drawn

General tool evaluating general key constructs underpinning measures						
Original checklist statement incorporated from<br><br>*1= COSMIN checklist (Mokkink, 2018)*<br><br>*2= Evidence Based Medicine and Practice (Roever & PE, 2015)*<br><br>*3= Joanna Briggs Checklist for Prevalence Studies (Joanna Briggs Institute, 2016)*<br><br>*4=Quantitative SLR checklist University of* Manchester Woods et al., 2011)			R1	R2	Quality Rating<br><br>*2 stars (**) indicates this was done well or in detail, 1 star (*) indicates this was done partially, hyphen (-) indicates unclear or incomplete processes*	Evidence in article
			Y= present<br>*N= not present*			
Development	1/2	Is a definition of the construct given?				
	2	Are research questions outlined?				

	1/3	Is a clear description provided of the target population for which the measure was developed?				
	1	Is the theory on which the measure is based outlined and explained?				
	1/2/ 3	Interviews conducted with children/adolescents regarding concept definition?				
	1/ 2	Was an appropriate qualitative data collection method used to identify relevant items for a measure?				
	1/3	Is an explanation provided to allow for replication?				
	1/3	Appropriate method used to analyse the data? (Numbers? Reductive?)				
Content validity/ Internal structure	1	Interviews conducted with experts regarding concept definition?				

	3	FA/structural equations model conducted at development stage? (bonus point)				
Internal consistency	1/3	Was Cronbach's alpha calculated above 0.8?				
	1/3	Invariance testing- Structural equation modelling in subsequent papers? (bonus point)				
Cross-cultural validity\measurement invariance	1	Did the authors consider variance across different groups and demonstrate understanding there may be variability in measure perception?				
Responsiveness (comparison to gold standard)	1/2	Does the author compare scores with variables expected to be related based on theory?				
	1	Are they suitable?				

Overall Quality Rating<br>*1-4 ** ratings = low quality paper*<br>*5-8 ** ratings = medium quality paper*<br>*10-13 ** ratings = high quality paper*	Number of two-star (**) ratings:		Qualitative rating:	

Table S2: Critical Appraisal Tool for Measure development papers and explanations of statements

Critical Appraisal statement		R1	R2	Quality Rating<br><br>*2 stars (**) indicates this was done well or in detail, 1 star (*) indicates this was done partially, hyphen (-) indicates unclear or incomplete processes*	Evidence from paper
		Y= present<br>*N= not present*			
Development	Core: Construct Definition<br>*Is a definition of the construct given? Clear reference to definition of loneliness being used*				
	Core: Research questions outlined<br>*Are research questions outlined?*				

	Core: Clear description of target population<br>*Is a clear description provided of the target population for which the measure was developed?*				
	Core: Theory outlined and described<br>*Is the theory on which the measure is based, outlined, and explained in detail?*				
	Core: Interviews conducted with children and/or adolescents<br>*Were interviews conducted with children/adolescents regarding concept definition? Part of the development process?*				
	Core: Data Collection method<br>*Was an appropriate qualitative data collection method used to identify relevant items for a measure? Interviews? Where did the items come from? Is this clearly explained?*				
	Core: Replication details included<br>*Is an explanation provided to allow for replication? Is it possible to replicate development procedures with details provided?*				
	Core: Appropriate data analysis<br>*Appropriate method used to analyse the data? Numbers? Reductive? Correlations?*				
Content validity/<br>Internal structure	Core: Interviews with experts regarding concept definition<br>*Interviews conducted with experts regarding concept definition? Inclusion of children/adolescents definition of loneliness?*				

	Supplementary expectation: Factor Analysis/ structural equations model<br>*Was an Exploratory or Confirmatory FA or structural equations model conducted at the development stage?*				
Internal consistency	Core: Good internal consistency<br>*Was Cronbach's alpha calculated above 0.8?*				
	Supplementary expectation: Invariance testing<br>*Structural equation modelling in subsequent papers?*				
Cross-cultural validity\measurement invariance	Core: Variance across different groups<br>*Did the authors consider cross-cultural validity during measure development and sampling? Cross-cultural validity? Measurement invariance using multi-group CFA?*				
Responsiveness (comparison to gold standard)	Core: Scores compared with related variables<br>*Compared with related variables? Suitably related?*				

	Core: Suitable comparisons<br>*Is it clear what the comparator instrument(s) measure(s)? Is there a good level of reliability for comparator instrument? Is there a good level of validity for comparator instrument?*<br>*Reference to concurrent validity?*					
Core: Overall quality decision<br>*1-4 ** ratings = low quality paper*<br>*5-8 ** ratings = medium quality paper*<br>*10-13 ** ratings = high quality paper*		Number of two-star (**) ratings:			Qualitative rating:	

Table S3: Completed Critical Appraisal Checklist Marcoen, Goossens & Caes (1987) (LACA)

Critical Appraisal statements	R1	R2	Quality Rating	Evidence from paper
	Y= present<br>*N= not present*		*2 stars (**) indicates this was done well or in detail, 1 star (*) indicates this was done partially, hyphen (-) indicates unclear or incomplete processes*	

Development	Core: Construct Definition<br>*Is a definition of the construct given? Clear reference to definition of loneliness being used*	Some	N	*	• "loneliness is though to ensure when the person's expectations regarding interpersonal relations cannot be met within his or her social network"<br>• "Directly asking for feelings of isolation and desertion in different kinds of relations, therefore, may provide a more adequate picture of loneliness"<br>• Describes Marcoen & Brumagne (1985)- children and adolescent measure"
	Research questions outlined<br>*Are research questions outlined?*	Y	Y	**	• "Main objectives…(a) to develop a psychometrically sounds, multidimensional loneliness measure; (b) to use this instrument in an exploration of age and sex differences in loneliness through late childhood and adolescence; and (c) to provide concurrent validity data for each of the subscales)
	Clear description of target population<br>*Is a clear description provided of the target population for which the measure was developed?*	Y	Y	**	• Children and adolescents<br>• "late childhood and adolescence"<br>• Clear description of ages of participants
	Theory outlined and described<br>*Is the theory on which the measure is based, outlined and explained in detail?*	Y	Y	**	• "From the beginning of scale development process, a need was felt to cover related constructs of positively and negatively experienced aloneness"<br>• Multidimensional instrument
	Interviews conducted with children and/or adolescents<br>*Were interviews conducted with children/adolescents regarding concept definition? Part of the development process?*	N	N	-	• Based on Marcoen & Brumagne (1985)- 2 subscales- parental relations and loneliness in peer relations and affinity for loneliness or aversion to loneliness<br>• No interviews

	Data Collection method *Was an appropriate qualitative data collection method used to identify relevant items for a measure? Interviews? Where did the items come from? Is this clearly explained?*	N	N	-	• Refer to Marcoen and Brumagne (1985) paper and completed checklist
	Replication details included *Is an explanation provided to allow for replication? Is it possible to replicate development procedures with details provided?*	N	N	-	• No explanation about how/why items chosen
	Appropriate data analysis *Appropriate method used to analyse the data? Numbers? Reductive?*	Y	Y	**	
Content validity/ Internal structure	Interviews with experts regarding concept definition *Interviews conducted with experts regarding concept definition? Inclusion of children/adolescents definition of loneliness?*	N	N	-	
	Supplementary expectation: Factor Analysis/ structural equations model *Was an Exploratory or Confirmatory FA or*	Y	Y	**	• Factor analysis conducted

	structural equations model conducted at the development stage?				
Internal consistency	Good internal consistency<br>*Was Cronbach's alpha calculated above 0.8?*	Y	Y	**	• Above 0.80
	Supplementary expectation: Invariance testing<br>*Structural equation modelling in subsequent papers?*	N	N	-	
Cross-cultural validity\measurement invariance		Some	Y	*	• Sex and age differences explored,<br>• No reference to cross-cultural differences
Responsiveness (comparison to gold standard)		Some	Some	*	• Compared to subjects' age, sex, parental occupation, 16 question<br>• 4 categories- social integration, home environment, ecological situation, psychological factors
		Some	Some	*	• Some comparison

Overall quality decision<br>*1-4 ** ratings = low quality paper*<br>*5-8 ** ratings = medium quality paper*<br>*10-13 ** ratings = high quality paper*	Number of two-star (**) ratings:	6	Qualitative rating:	Medium

Table S4: Completed Critical Appraisal Checklist Marcoen & Brumagne (1985) (LLCA)

Critical Appraisal statements		R1	R2	Quality Rating	Evidence from paper
		Y= present<br>*N= not present*		*2 stars (**) indicates this was done well or in detail, 1 star (*) indicates this was done partially, hyphen (-) indicates unclear or incomplete processes*	
Development	Core: Construct Definition<br>*Is a definition of the construct given? Clear reference to definition of loneliness being used*	Some	Some	-	• "Little or no empirical research… age-linked feelings of loneliness among children and adolescents"<br>• Definition from Weiss (1973)- makes distinction between emotional isolation and social isolation<br>• "We distinguished between loneliness in relation to parents, or parent-relate loneliness, and loneliness in relation to peers or peer related loneliness".
	Research questions outlined<br>*Are research questions outlined?*	N	N	-	• "the relation of loneliness to another aspect of social status, namely, perceived social sensitivity, was examined in the present research"

					• "No specific prediction with regard to the relation between perceived social sensitivity and parent-related loneliness were formulated"<br>• Research aims not clearly outlined
	Clear description of target population<br>*Is a clear description provided of the target population for which the measure was developed?*	Y	Y	**	• Outlines ages of participants<br>• Refers to children and adolescents on several occasions
	Theory outlined and described<br>*Is the theory on which the measure is based, outlined and explained in detail?*	Some	Some	*	• Refers to previous research on loneliness in children and adolescents<br>• Makes distinctions between emotional isolation and social isolation<br>• Family and peer group
	Interviews conducted with children and/or adolescents<br>*Were interviews conducted with children/adolescents regarding concept definition? Part of the development process?*	N	N	-	• No reference to interviews with children/adolescents
	Data Collection method<br>*Was an appropriate qualitative data collection method used to identify relevant items for a measure? Interviews? Where did the items come from? Is this clearly explained?*	N	N	-	• "original scale"

	Replication details included *Is an explanation provided to allow for replication? Is it possible to replicate development procedures with details provided?*	N	N	-	
	Appropriate data analysis *Appropriate method used to analyse the data? Numbers? Reductive?*	Y	N	**	
Content validity/ Internal structure	Interviews with experts regarding concept definition *Interviews conducted with experts regarding concept definition? Inclusion of children/adolescents definition of loneliness?*	N	N	-	• No reference to interviews
	Supplementary expectation: Factor Analysis/ structural equations model *Was an Exploratory or Confirmatory FA or structural equations model conducted at the development stage?*	Y	Y	**	• FA conducted
Internal consistency	Good internal consistency *Was Cronbach's alpha calculated above 0.8?*	Y	N	**	• .88

	Supplementary expectation: Invariance testing<br>*Structural equation modelling in subsequent papers?*	N	N	-	
Cross-cultural validity\measurement invariance	Variance across different groups<br>*Did the authors consider cross-cultural validity during measure development and sampling?*	Some	Some	*	• Catholic schools in two Dutch-speaking Flemish towns in Belgium<br>• Sex, Age (Table 1 and 2)
Responsiveness (comparison to gold standard)	Scores compared with related variables<br>*Compared with related variables? Suitably related?*	Some	Some	*	Syracuse-Amsterdam- Groningen Sociometric Scale (SAGS) to determine first-comfort figures and measure perceived social sensitivity. "Each subject was evaluated by his or her classmates with respect to his or her being a source of comfort, support and sympathy in periods of unhappiness and sadness"
	Suitable comparisons<br>*Is it clear what the comparator instrument(s) measure(s)? Is there a good level of reliability for comparator instrument? Is there a good level of validity for comparator instrument?*	N	N	*	
Overall quality decision<br>*1-4 ** ratings = low quality paper*<br>*5-8 ** ratings = medium quality paper*<br>*10-13 ** ratings = high quality paper*				Number of two-star (**) ratings: 4	Qualitative rating: Low

Table S5: Completed Critical Appraisal Checklist Russell, Peplau &Ferguson (1978) (UCLA)

Critical Appraisal statement		R1	R2	Quality Rating	Evidence from paper
		Y= present N= not present		*2 stars (**) indicates this was done well or in detail, 1 star (*) indicates this was done partially, hyphen (-) indicates unclear or incomplete processes*	
Development	Core: Construct Definition<br>*Is a definition of the construct given? Clear reference to definition of loneliness being used*	N	N	-	"Loneliness is a condition that is widely distributed and severely distressing"
	Research questions outlined<br>*Are research questions outlined?*	Y	Y	*	"The present article report the development of a short an highly reliable general loneliness scale that appears t have concurrent and construct validity, based on several criteria"<br>No reference to who it is for
	Clear description of target population<br>*Is a clear description provided of the target population for which the measure was developed?*	N	N	-	• "undergraduate students in introductory psychology classes"<br>• Sample age not defined clearly
	Theory outlined and described<br>*Is the theory on which the measure is based, outlined and explained in detail?*	N	N	-	• Talks generally about research loneliness and lack of simple methods of assessment.

	Interviews conducted with children and/or adolescents<br>*Were interviews conducted with children/adolescents regarding concept definition? Part of the development process?*	N	N	-	• No mention of children/adolescents<br>• No age of participants given<br>• “Undergraduate students”<br>• No development info given
	Data Collection method<br>*Was an appropriate qualitative data collection method used to identify relevant items for a measure? Interviews? Where did the items come from? Is this clearly explained?*	N	N	-	• Items based on Sisenwein (1964) paper- “20 psychologists describing the experience of loneliness”. And statements from Eddy’s (1961) paper- unpublished doctoral thesis.<br>• “Items were selected to preserve diversity yet exclude very extreme statements”
	Replication details included<br>*Is an explanation provided to allow for replication? Is it possible to replicate development procedures with details provided?*	Some	Some	*	• Refers to 75 items that statements were selected from. No information on which from Sisenwein/Eddy
	Appropriate data analysis<br>*Appropriate method used to analyse the data? Numbers? Reductive?*	Y	Y	**	• Alpha, correlations between “subjective self-report question about current loneliness and the loneliness scale score”, Beck depression scale and self-reported ratings of “depressed” and “anxious” test retest
Content validity/ Internal structure	Interviews with experts regarding concept definition<br>*Interviews conducted with experts regarding concept definition? Inclusion of*	N	N	-	• Sisenwein- psychologist statements- but are they experts? Is their idea of loneliness the same as college students studying psychology?

	children/adolescents definition of loneliness?				
	Supplementary expectation: Factor Analysis/ structural equations model<br>*Was an Exploratory or Confirmatory FA or structural equations model conducted at the development stage?*	N	N	-	• Correlations between items and overall score
Internal consistency	Good internal consistency<br>*Was Cronbach's alpha calculated above 0.8?*	Y	Y	**	• Above 0.80 and 2-month retest
	Supplementary expectation: Invariance testing<br>*Structural equation modelling in subsequent papers?*	N	N	-	
Cross-cultural validity\measurement invariance	Variance across different groups<br>*Did the authors consider cross-cultural validity during measure development and sampling?*	Some	Some	*	• Examined by region and sex<br>• Table 1- UCLA/Tulsa Sample<br>• Males/Females

Responsiveness (comparison to gold standard)	Scores compared with related variables<br>*Compared with related variables? Suitably related?*	Some	Some	*		• Correlation emotion/ emotional states<br>Correlation between UCLA score and each item<br>• Problematic?	
	Suitable comparisons<br>*Is it clear what the comparator instrument(s) measure(s)? Is there a good level of reliability for comparator instrument? Is there a good level of validity for comparator instrument?*	Some	Some	*		• Are the ratings of "depressed" and "anxious" a direct comparison to loneliness?	
Overall quality decision<br>*1-4 ** ratings = low quality paper*<br>*5-8 ** ratings = medium quality paper*<br>*10-13 ** ratings = high quality paper*				Number of two-star (**) ratings:	2	Qualitative rating:	Low

Table S6: Completed Critical Appraisal Checklist CLS

Critical Appraisal statements		R1	R2	Quality Rating	Evidence from paper
		Y= present N= not present		*2 stars (**) indicates this was done well or in detail, 1 star (*) indicates this was done partially, hyphen (-) indicates unclear or incomplete processes*	
Development	Core: Construct Definition<br>*Is a definition of the construct given? Clear reference to definition of loneliness being used*	Y	Y	**	• One limitation of the intervention literature has been the absence of information concerning unpopular children's perspective about their own situation"
	Research questions outlined<br>*Are research questions outlined?*	Y	Y	**	• "The goals of the present research were to develop a reliable measure of children's feelings of loneliness and social dissatisfaction and to learn whether children who are least accepted by their classmates are indeed more lonely"
	Clear description of target population<br>*Is a clear description provided of the target population for which the measure was developed?*	Y	Y	**	• "Children" particularly least accepted by peers
	Theory outlined and described<br>*Is the theory on which the measure is based, outlined and explained in detail?*	Some	Some	*	• Some reference to previous research<br>• Not provide exact theory<br>• "may intervention studies with unpopular children use sociometric measures to select children who are least liked in their classroom" "one limitation has been the absence of information concerning unpopular children's perspective about their own situation"

	Interviews conducted with children and/or adolescents *Were interviews conducted with children/adolescents regarding concept definition? Part of the development process?*	N	N	-	• "a 24-item questionnaire was developed to assess children's feelings of loneliness and social dissatisfaction"- no reference to where items came from
	Data Collection method *Was an appropriate qualitative data collection method used to identify relevant items for a measure? Interviews? Where did the items come from? Is this clearly explained?*	N	N	-	• Questions developed by researchers?
	Replication details included *Is an explanation provided to allow for replication? Is it possible to replicate development procedures with details provided?*	N	N	-	• No details about how items were developed
	Appropriate data analysis *Appropriate method used to analyse the data? Numbers? Reductive?*	Y	Y	**	• Factor analysis conducted
Content validity/ Internal structure	Interviews with experts regarding concept definition *Interviews conducted with experts regarding concept definition? Inclusion of*	N	N	-	• No reference to experts (children) being consulted in construction

	children/adolescents definition of loneliness?				
	Supplementary expectation: Factor Analysis/ structural equations model *Was an Exploratory or Confirmatory FA or structural equations model conducted at the development stage?*	Y	Y	**	• Factor analysis conducted (Table 3)
Internal consistency	Good internal consistency *Was Cronbach's alpha calculated above 0.8?*	Y	Y	**	• Above .80
	Supplementary expectation: Invariance testing *Structural equation modelling in subsequent papers?*	N	N	-	
Cross-cultural validity\measurement invariance	Variance across different groups *Did the authors consider cross-cultural validity during measure development and sampling?*	N	N	-	

<table>
<tr><td rowspan="2">Responsiveness (comparison to gold standard)</td><td>Scores compared with related variables
Compared with related variables? Suitably related?</td><td>Some</td><td>Some</td><td colspan="2">*</td><td colspan="2">• Sociometric measures, loneliness and sociometric status- positive nomination measure and rating-scale measure to rate each classmate
• Notes issue…
• “we considered whether children who might be targeted for intervention on the basis of sociometric measures reported greater loneliness and social dissatisfaction than their higher-status peers”
• Second analysis was conducted to examine whether children with few or no best friendship nominations within their classroom would experience greater loneliness”</td></tr>
<tr><td>Suitable comparisons
Is it clear what the comparator instrument(s) measure(s)? Is there a good level of reliability for comparator instrument? Is there a good level of validity for comparator instrument?</td><td>Some</td><td>Some</td><td colspan="2">*</td><td colspan="2">• Is there a close comparison between loneliness and dissatisfaction and sociometric status?</td></tr>
<tr><td colspan="4">Overall quality decision
1-4 ** ratings = low quality paper
*5-8 ** ratings = medium quality paper*
*10-13 ** ratings = high quality paper*</td><td>Number of two-star (**) ratings:</td><td>6</td><td>Qualitative rating:</td><td>Medium</td></tr>
</table>
